# Murine Typhus Presenting as Septic Acute Cholangitis in a Young Woman From South Texas

**DOI:** 10.7759/cureus.19209

**Published:** 2021-11-02

**Authors:** Jeffrey Xia, Sabi Shrestha, James C Saca

**Affiliations:** 1 Department of Medicine, University of Texas Health Science Center at San Antonio, San Antonio, USA

**Keywords:** cholestatic, elevated liver transaminases, hepatology, ercp, eus, cholecystitis, cholangitis, sepsis, rickettsia, murine typhus

## Abstract

Murine (endemic) typhus is a zoonotic disease spread by fleas carrying *Rickettsia typhi* bacteria. Typically, murine typhus presents with mild and nonspecific flu-like symptoms. However, it can manifest with severe systemic complications potentially leading to delayed treatment or unnecessary interventions.

We present the case of a young woman from South Texas who presented to the emergency department after 10 days of fever, myalgia, headache, nausea, and right-sided abdominal pain. She was found to be febrile, severely hypotensive, suffering from acute liver injury with a predominantly cholestatic pattern, acute kidney injury, severe thrombocytopenia, and hyponatremia. She was initially managed with broad-spectrum antibiotics for undifferentiated septic shock, and doxycycline was added due to suspicion of a *Rickettsial* infection. Although radiographic findings showed some evidence of biliary involvement, they were not typical for common biliary diseases. However, due to her severe clinical presentation and findings suggesting possible acute cholangitis, she underwent an endoscopic ultrasound with endoscopic retrograde cholangiopancreatography, which revealed no evidence of acute obstructive biliary disease. Without strong evidence to explain her presentation, an extensive chronic liver disease workup was done, which was negative. The patient ultimately clinically improved with antibiotics alone.

This case demonstrates an atypical presentation of murine typhus, presenting with septic shock and masquerading as acute cholangitis. With the rising incidence of murine typhus in endemic areas of the United States, this case reinforces the importance of being cognizant of the typical and atypical presentations of murine typhus, which may allow for early appropriate treatment and potentially avoid unnecessary interventions. Additionally, in this study, we conducted a literature review of murine typhus cases associated with acute biliary dysfunction.

## Introduction

Murine typhus has a worldwide distribution. Although epidemic and endemic (murine) typhus have almost been eliminated from the United States, most cases of murine typhus are currently concentrated in endemic areas such as Texas and California [1]. Murine typhus is caused by *Rickettsia typhi*, a gram-negative, intracellular, rod-shaped bacterium transmitted by rat or cat flea vectors. This condition often presents with nonspecific and mild symptoms such as fever, myalgia, and rash. Rarely, murine typhus presents with atypical or severe symptoms resulting in a challenging diagnosis [1,2]. The condition is likely underdiagnosed due to its nonspecific clinical presentation, frequently unclear history of flea exposure, and the possibility of spontaneous resolution without medical treatment. Here, we present a case that illustrates a rare presentation of murine typhus presenting as septic shock while masquerading as acute cholangitis.

## Case presentation

A 40-year-old Caucasian woman from South Texas with a medical history of hypothyroidism, type 2 diabetes, and hypertension presented to her primary care physician with five days of intermittent fever, chills, headache, cough, myalgia, nausea, and cervical lymphadenopathy in the month of July. She was given a methylprednisolone injection and prescriptions for cefdinir and ondansetron for presumed bacterial upper respiratory tract infection and symptomatic management. She had partial relief of symptoms for approximately one day, but the fever resumed, peaking at 104°F. At this point, she developed new and persistent mid-epigastric and right-sided abdominal pain and a rash on her back. She attempted to manage her symptoms with supportive care for four more days but was unable to tolerate the symptoms and presented to the emergency department.

**Figure 1:**
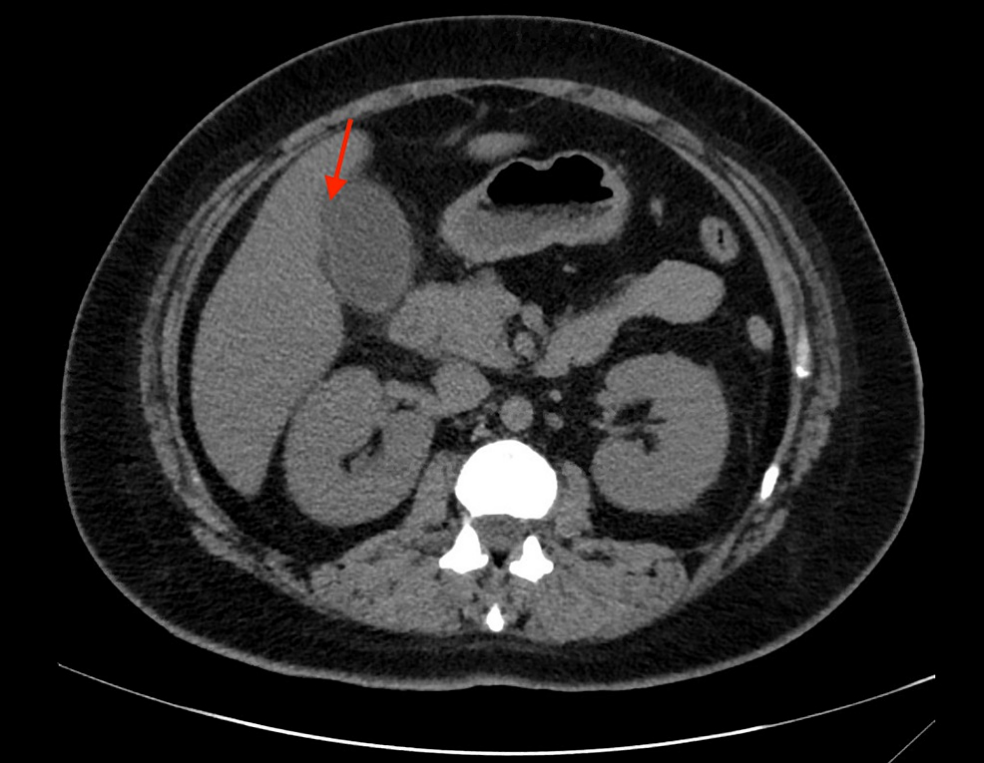
Without-contrast CT of the abdomen/pelvis of a patient suspected to have biliary obstruction, who was later found to have murine typhus. The red arrow points to subtle pericholecystic fat stranding. CT = computed tomography

In the emergency department, she was found to be severely hypotensive (73/56 mmHg) and febrile (101.4°F). Despite intravenous fluid resuscitation, her blood pressure did not improve, and a norepinephrine drip was initiated. Vancomycin and piperacillin-tazobactam were started as empiric treatment for undifferentiated septic shock. Physical examination demonstrated right upper quadrant (RUQ) tenderness to palpation and slight maculopapular rash on the trunk. Preliminary laboratory investigations were significant for acute kidney injury, acute liver injury with predominantly cholestatic pattern, mildly elevated transaminases, moderate hyponatremia, hypoalbuminemia, severe thrombocytopenia, and mild leukocytosis (Table 1). Serum *Rickettsia typhi* immunoglobulin (Ig)M and IgG were collected. At this point, murine typhus was suspected to be part of the clinical picture, but her only interaction with flea-carrying animals was occasionally leaving food bowls for stray cats. CT abdomen was significant only for subtle pericholecystic fat stranding without gallstones (Figure 1). RUQ ultrasound revealed normal common bile duct (CBD), mild gallbladder wall thickening, and mildly increased liver echogenicity. Due to her severe presentation, she was admitted to the intensive care unit (ICU).

**Table 1:** Laboratory data on admission. aPTT = activated partial thromboplastin time; INR = international normalized ratio

Variable	Reference range	On admission
Sodium (mmol/L)	135–145	129
Potassium (mmol/L)	3.5–5.1	4.1
Blood urea nitrogen (mg/dL)	7–25	49
Creatinine (mg/dL)	0.50–1.10	3.31
Alkaline phosphatase (U/L)	45–117	450
Alanine aminotransferase (U/L)	<36	137
Aspartate aminotransferase (U/L)	<32	72
Total bilirubin (mg/dL)	0.2–1.2	5.2
Protein		
Total (g/dL)	6.2–8.1	5.8
Albumin (g/dL)	3.2–5.0	2.3
Lactic acid (mmol/L)	0.5–2.0	5.3
D-dimer (ng/mL)	<500	29,184
Fibrinogen (mg/dL)	152–445	404
aPTT (seconds)	25–37	36
INR	0.8–1.2	1.1
White blood cell count (K/µL)	3.40`10.40	11.35
Differential count (K/µL)		
Neutrophils	1.50–6.60	10.67
Lymphocytes	0.90–3.60	0.57
Monocytes	0.20–1.30	0.11
Eosinophils	<0.40	0.00
Basophils	<0.10	0.00
Hemoglobin (g/dL)	11.5–14.9	12.3
Hematocrit (%)	36.0–45.5	35.8
Platelet count (K/µL)	140–377	32
Erythrocyte sedimentation rate (mm/hour)	2–37	44
C-reactive protein (mg/L)	0.00–10.00	398.50

**Figure 2:**
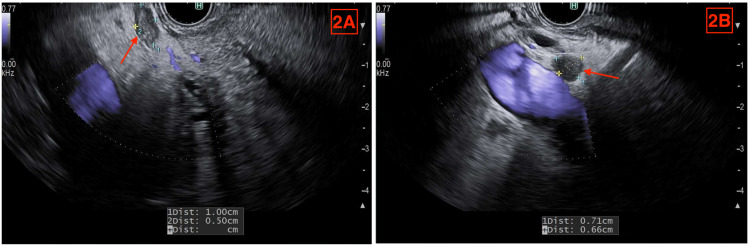
Two mildly enlarged periportal lymph nodes discovered on EUS in a patient suspected to have biliary obstruction, who was later found to have murine typhus. Red arrows point to the lymph nodes. (A) Lymph node measuring 10 mm × 5 mm. (B) Lymph node measuring 7.1 mm × 6.6 mm. EUS = endoscopic ultrasound

In the ICU, she was stabilized and tolerated a test dose of doxycycline because she had a reported minocycline allergy. Antibiotics were adjusted to include piperacillin-tazobactam with 100 mg doxycycline tablet twice daily. A hepatobiliary iminodiacetic acid (HIDA) scan was positive, showing absent gallbladder activity at 4.5 hours. Due to her clinical presentation and radiographic findings, general surgery was consulted. General surgery was most suspicious for acute cholangitis and recommended urgent endoscopic retrograde cholangiopancreatography (ERCP). The endoscopic ultrasound (EUS) revealed a CBD diameter of 4.5 mm, mild gallbladder wall thickening, and two mildly enlarged, reactive periportal lymph nodes measuring 7.1 mm × 6.6 mm and 10.0 mm × 5.0 mm (Figure 2). The ERCP demonstrated patent and normal functioning CBD and cystic duct with no gallstones. Sphincterotomy was completed despite no evidence of biliary obstruction. Hepatology was consulted due to the lack of clear endosonographic findings to explain her clinical findings.

On post-admission day three, the patient defervesced, remained normotensive, and was downgraded from the ICU. Serum transaminases continued to rise (aspartate aminotransferase [AST]: 155 U/L, alanine aminotransferase [ALT]: 110 U/L), total bilirubin decreased slightly (4.6 mg/dL), and alkaline phosphatase levels remained elevated (457 U/L). Serologies recommended by Hepatology were nonreactive/negative to hepatitis A, B, and C virus, human immunodeficiency virus, herpes simplex virus, rapid plasma reagin, parvovirus B19, Epstein-Barr virus, cytomegalovirus, *Ehrlichia*, anti-mitochondrial antibodies, anti-smooth muscle antibodies, and anti-liver/kidney microsome antibodies, with normal levels of alpha-1 antitrypsin, ceruloplasmin, ammonia, and IgG subclasses. The patient had a positive anti-nuclear antibody (ANA) titer (>1:640) which was previously known to be positive and deemed to have no clinical significance after prior workup.

On post-admission day four, *Rickettsia typhi* IgM titer was positive at 1:256 with an IgG titer of <1:64. At this point, AST and ALT continued to rise and peaked at 167 U/L and 139 U/L, respectively. Hepatology recommended a liver biopsy to investigate the continued and unexplained rising levels of serum transaminases in the context of positive ANA titer. The liver biopsy showed mixed lymphoplasmacytic mononuclear and neutrophilic infiltrate in portal tracts with minimal expansion, slightly reactive hepatocytes throughout with scattered inflammatory cells, and mild focal hepatocyte ballooning seen in zone three. Four blood cultures from days one and two remained negative. She was discharged on post-admission day six symptom-free with down-trending serum transaminases on doxycycline and follow-up with outpatient Hepatology.

## Discussion

Murine typhus was first identified in the United States in 1913 when its incidence was more than 5,000 cases per year. In 1945, a major effort by the US Public Health Service to control rat populations decreased the annual incidence to fewer than 100 cases [1]. Murine typhus was last a nationally notifiable disease in 1987 with 49 incident cases in the United States; however, it remains a state-notifiable disease in some states such as Texas and California, where the most reported cases of murine typhus occur in the country [3]. The incidence of reported murine typhus cases has increased over the past decade. From 2010 to 2018, cases of reported murine typhus in Texas increased from 135 to 738, while cases in California increased from 54 to 174 in the same time interval [4,5].

Typically, murine typhus presents with an abrupt onset of nonspecific symptoms, including fever, headache, myalgia, chills, and a macular or maculopapular rash over the trunk [6,7]. These vague symptoms often result in the initial misdiagnosis of murine typhus. Basic laboratory findings may aid in the diagnosis of murine typhus, including serum liver transaminase elevation, lactate dehydrogenase elevation, thrombocytopenia, and hyponatremia [6,7]. It is typically a mild, self-limiting disease. A recent systematic review found that 26.1% of patients with murine typhus developed complications: more commonly, pneumonia/pulmonary infiltrates, altered mental status, and acute kidney injury. Among those who had complications, 5.9% required ICU care and 0.8% developed septic shock/multiorgan failure [6].

This case illustrates the potential of murine typhus to present similar to acute cholangitis and the diagnostic challenge of the atypical presentations of murine typhus. Our patient’s clinical presentation and laboratory findings (RUQ tenderness, fever, hyperbilirubinemia) were initially suggestive of acute cholangitis, but there was weak radiographic evidence of biliary dilatation or obstruction on CT and ultrasound. At one point, acute cholecystitis was considered due to the positive HIDA scan, but general surgery prioritized acute cholangitis due to the patient’s overall clinical findings, hyperbilirubinemia, and increased false-positive rate of HIDA scan with severe illness, anorexia, and abnormal hepatocellular function [8]. However, acute cholangitis was ruled out after ERCP due to the absence of gallstones, CBD stone, biliary dilatation on EUS, and purulent material after ERCP balloon sweeping. Even though the ERCP was nontherapeutic, the patient eventually clinically improved on doxycycline. Due to this sequence of events and subsequent result of positive *Rickettsia typhi *IgM, this case is an atypical presentation of murine typhus mimicking acute cholangitis. Our patient received an extensive chronic liver disease workup and liver biopsy which could have been avoided given that these investigations ultimately did not provide additional useful information.

It is not uncommon for murine typhus to mimic other disease states including acute cholecystitis. A 52-year-old man in Thailand who presented with fever, RUQ tenderness, and hypotension was suspected of having septic acute cholecystitis and underwent a cholecystectomy. No stone was found, and he was later serologically diagnosed with murine typhus [9]. A liver biopsy was performed which showed infiltration of portal tracts and central vein regions with neutrophils and lymphocytes, cloudy swelling of hepatocytes, prominent mitoses, similar to the liver biopsy findings of our patient [9].

In addition to atypical presentations, it is important to understand that there are reported cases of acute cholecystitis occurring as a bonafide complication with murine typhus. A 54-year-old man from Greece who presented with a 10-day history of fever, malaise, and headache developed RUQ tenderness and elevated serum transaminases with sonographic findings of a thickened gallbladder, mild pericholecystic fluid, and positive Murphy’s sign. He was diagnosed with acalculous cholecystitis and murine typhus and was appropriately treated with doxycycline [10]. A 51-year-old Australian man who traveled to Asia presented to the hospital in a febrile condition with RUQ tenderness and radiographic findings consistent with acute cholecystitis. He was able to avoid cholecystectomy after positive serologies for *Rickettsia typhi*, following which he was appropriately treated with doxycycline [11].

Furthermore, in a case series study by Afzal et al. involving 90 hospitalized patients with murine typhus in South Texas, 28% of the cohort developed complications, with one patient developing cholecystitis. The results of this study suggest that among hospitalized patients with murine typhus, complication rate is fairly high, but the involvement of the gallbladder and biliary system is rare [12].

## Conclusions

This case exhibits an atypical presentation of murine typhus mimicking acute cholangitis. In endemic areas, it is important to consider murine typhus on the differential diagnosis for undifferentiated febrile illnesses acquired from the community because of the possibility of complications related to delayed treatment. Early recognition and management of murine typhus may prevent unnecessary diagnostic and surgical interventions. This case illustrates that a strong exposure history for murine typhus is not always clear. Maintaining a high index of suspicion in endemic areas and knowledge of atypical and severe presentations allow for early initiation of therapy, which generally leads to rapid clinical improvement.
